# The cytochrome P450 (CYP) superfamily in cnidarians

**DOI:** 10.1038/s41598-021-88700-y

**Published:** 2021-05-10

**Authors:** Kirill V. Pankov, Andrew G. McArthur, David A. Gold, David R. Nelson, Jared V. Goldstone, Joanna Y. Wilson

**Affiliations:** 1 M.G. DeGroote Institute for Infectious Disease Research, Department of Biochemistry and Biomedical Sciences, DeGroote School of Medicine, McMaster University, Hamilton, ON Canada; 2 Department of Biology, McMaster University, 1280 Main Street West, Hamilton, ON L8S 4K1 Canada; 3 Department of Earth and Planetary Sciences, University of California Davis, Davis, CA USA; 4 Department of Microbiology, Immunology and Biochemistry, University of Tennessee, Memphis, TN USA; 5 Department of Biology, Woods Hole Oceanographic Institution, Woods Hole, MA USA

**Keywords:** Phylogeny, Enzymes

## Abstract

The cytochrome P450 (CYP) superfamily is a diverse and important enzyme family, playing a central role in chemical defense and in synthesis and metabolism of major biological signaling molecules. The CYPomes of four cnidarian genomes (*Hydra vulgaris*, *Acropora digitifera*, *Aurelia aurita*, *Nematostella vectensis*) were annotated; phylogenetic analyses determined the evolutionary relationships amongst the sequences and with existing metazoan CYPs. 155 functional CYPs were identified and 90 fragments. Genes were from 24 new CYP families and several new subfamilies; genes were in 9 of the 12 established metazoan CYP clans. All species had large expansions of clan 2 diversity, with *H. vulgaris* having reduced diversity for both clan 3 and mitochondrial clan. We identified potential candidates for xenobiotic metabolism and steroidogenesis. That each genome contained multiple, novel CYP families may reflect the large evolutionary distance within the cnidarians, unique physiology in the cnidarian classes, and/or different ecology of the individual species.

## Introduction

### The cytochrome P450 superfamily

The cytochrome P450 (CYP) superfamily consists of a large group of hemeproteins that catalyze a wide range of reactions, playing important roles in several fundamental biological processes (e.g. steroid synthesis, fatty acid metabolism, chemical defense)^1^. CYPs primarily perform a monooxygenase function, adding polar hydroxyl groups to a substrate, making it more reactive and hydrophilic^2^. Each individual CYP has a specific suite of compounds that fit into its active site, often with a structure–activity relationship. Collectively, CYP enzymes interact with both endogenous (e.g. steroids and fatty acids) and exogenous (e.g. drugs, plant secondary metabolites, and pollutants) compounds with highly diverse chemical structures. Thus, the CYP superfamily plays a role in multiple, critical physiological processes. In vertebrates, the CYP11, CYP17, CYP19, CYP21 and CYP51 families catalyze steps in the synthesis of hormones, including sex steroids^3^. The CYP51 family, the only CYP family found in all domains of life, produces an intermediate, 4,4-dimethylcholesta-8(9),14,24-trien-3β-ol, in the cholesterol biosynthesis pathway^4^, the end product of which is an important component of the lipid bilayer and involved in cell signaling. Many of the enzymes in the CYP4 family play a role in the metabolism of omega fatty acids^5^, providing a source of adenosine triphosphate (ATP) for cells when carbohydrate stores are low. Certain proteins in the CYP1, CYP2, and CYP3 families have high substrate specificity for foreign compounds such as polycyclic aromatic hydrocarbons (PAHs) and drugs, modifying them as part of xenobiotic response and detoxification^6,7^.

CYP genes are named according to a standard set of nomenclature conventions based largely on sequence similarity^8,9^. CYPs with greater than 40% amino acid sequence identity belong to the same family (represented by a number) and those with greater than 55% sequence identity belong to the same subfamily (represented by a letter). Each CYP is assigned a gene number, typically based on order of discovery. As an example, CYP17A1 would be the first gene identified in the ‘A’ subfamily of the ‘CYP17′ family. A broader classification of CYPs into ‘clans’ has also been implemented, based on phylogenetic relationships among CYP families^10^. All of the CYP families that repeatedly cluster in the same phylogenetic clade are grouped into the same clan, which is usually given the name of the smallest number family present in that clade. For example, since CYP39, CYP7 and CYP8 sequences consistently form a single clade in phylogenetic analyses, all three families became part of clan 7.

The crucial nature of CYP proteins is emphasized by their presence in some capacity across all domains of life and they are thought to be present in all metazoan species^11^. The progression of genomic sequencing technologies has allowed the entire CYP gene complement (the CYPome) of many metazoan species to be identified, including those from major metazoan phyla. Studies have been completed on chordates^7^, model organisms such as mouse, fish, frog, and chicken, and fish such as *Danio rerio* (zebrafish)^12^ and *Takifugu rubripes* (fugu pufferfish)^13^. Other phyla that have been investigated in detail include the hemichordate *Ciona intestinalis* (sea squirt tunicate)^14^, echinoderm *Stronglyocentrotus purpuratus* (sea urchin)^15^, the arthropods *Drosophila melanogaster* (fruit fly)^16^ and *Daphnia pulex* (water flea crustacean)^17^, and the annelid *Capitella teleta* (marine worm)^18^. In many of these studies, full length and partial genes have been identified (as well as pseudogenes), along with estimations of intron–exon boundaries. In general, most vertebrate genomes tend to have between 50–100 CYP genes^19^, but the number of genes expected in other metazoan species is hard to predict as the diversity and evolutionary origins of individual CYP families is not well understood. For example, the genome of the annelid *Capitella teleta* contained 24 novel CYP families and 7 novel CYP subfamilies amongst its predicted total of 96 functional CYPs^18^. We can anticipate continued discovery of novel CYP genes where function is completely unknown as we sequence an increasingly broad diversity of metazoan genomes. While the number of novel CYPs varies across phyla (even the human genome contains the poorly understood “orphan” CYP20 enzyme)^20^, they are of great interest as identification of their function may provide important clues to the unique physiology and environmental stressors of different species, as well as the evolution of core physiological processes such as steroid biosynthesis.

Relatively little work has been done to identify cytochrome P450s in the species that comprise the cnidarian phylum, one of the most diverse and earliest-branching groups on the metazoan tree of life. One defensome study identified CYP sequences from the starlet sea anemone *Nematostella vectensis*, but the 82 genes were not named, in part due to their low similarity with known CYP genes^21^. With more recent genome sequencing in other cnidarians, notably the moon jellyfish *Aurelia aurita*^22^ and brown hydra *Hydra vulgaris*^23^ and stony coral *Acropora digitfera*^24^, we now have an opportunity to examine cnidarian CYPomes in detail.

### The cnidarian phylum: evolutionary significance

**Figure 1:**
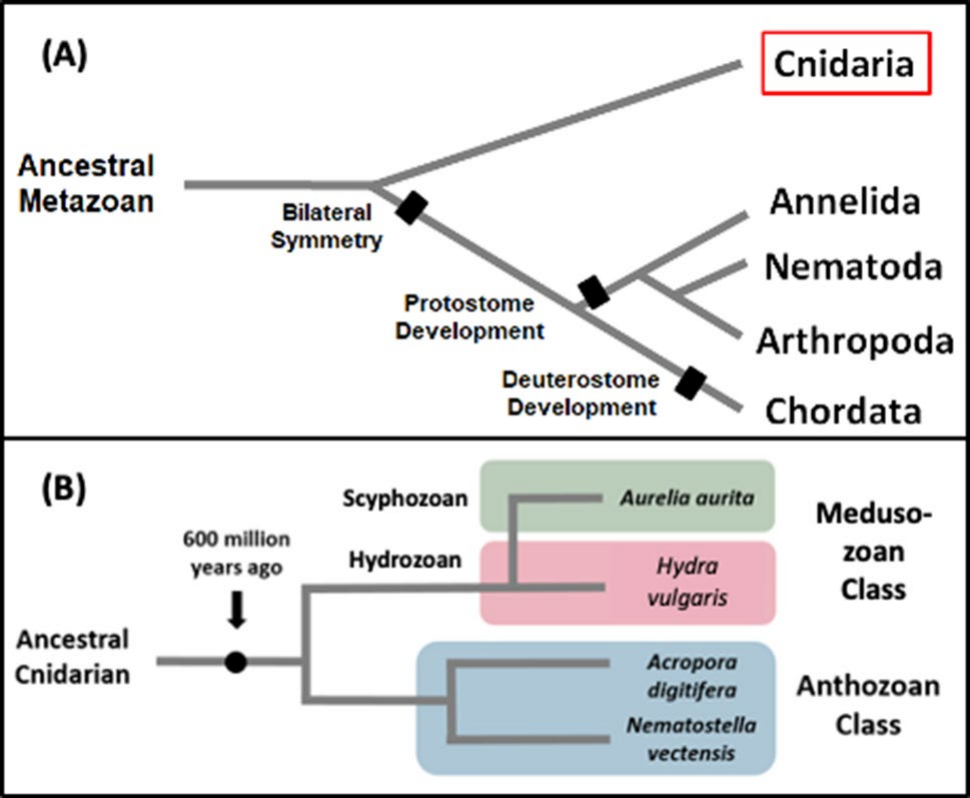
A simplified view of the cnidarian phylum in the tree of life. Branch lengths on the trees are arbitrary and meant only to visually display the relationship between the clades of species, not evolutionary distance between them. (**A**) The placement of the cnidarian phylum on the metazoan tree of life is highlighted. It diverged from the majority of metazoans, before the development of bilateral symmetry and the split between protostome and deuterostome development. (**B**) Cartoon of the relationships within the cnidarian phylum. The placement of *A. aurita*, *H. vulgaris*, *A. digitifera* and *N. vectensis* is illustrated. Adapted from^25,30^.

There are more than 20,000 known cnidarian species, including many types of jellyfish, corals, anemones and other aquatic animals. Many of these species have become cost-effective models for research related to toxicology, regeneration and other biochemical systems^25^. Evidence from ribosomal DNA indicates that the common ancestor of the cnidarian phylum diverged from the rest of the metazoans approximately 600 million years ago^26^; although more recent molecular studies support an older divergence in the Neoproterozoic Era, around 600–800 million years ago^27–29^. This was before the split between protostomes (arthropods, annelids and mollusks) and deuterostomes (chordates, hemichordates, and echinoderms) that defines many of the species that have had their CYP content characterized (Fig. 1A). The lack of data on CYP genes from this area of the tree of life is a major gap in understanding CYP evolution in metazoans, and the functional role that CYPs may play in cnidarian physiology. There is extensive morphological, behavioural, developmental, and ecological diversity within the cnidaria and an understanding of CYP genes in the context of generation of biological signaling molecules and metabolism may be highly relevant to the diversity within this ancient lineage. The defining feature that unites the phylum is the presence of a specialized cell known as a cnidocyte, often referred to as the ‘stinging cell’. It produces a structure called a cnida (i.e. nematocyst) that contains toxins used as defense against predators and as a means of attack to capture prey^25^. Within the phylum, the three major lineages are the anthozoa, endocnidozoa and medusozoa; hydrozoans, scyphozoans and cubozoans are all in the medusozoa^30^. The anthozoan class is the most abundant in species and includes corals and anemones that reside on marine seafloors and act as habitats for other aquatic animals. The endocnidozoa consist of parasitic species. The medusozoan are the jellyfish, hydroids and siphonophores. Hydrozoans such as the brown hydra are small predatory animals that often live in salt and freshwater habitats, typically in large colonies. The scyphozoans (i.e. “true jellies”) and cubozoans (i.e. box jellyfish) play important roles in the pelagic food chain and ecology. Cnidarian life cycles can include swimming medusae and sessile polyp stages, asexual and/or sexual reproduction, free-living and parasitic stages, solitary or colonial groupings, and associations with photosynthetic dinoflagellates.

Complete genome assemblies had been published for several cnidarian species; we selected genomes from two of the three cnidarian classes: anthozoa (*Nematostella vectensis*, *Acropora digitifera*) and medusozoan (the hydrozoan *Hydra vulgaris* and the scyphozoan *Aurelia aurita)* (Fig. 1B)^25^; the *Clytia* genome has also recently been completed^31^. The hydrozoan *H. vulgaris*, commonly known as the brown hydra, has been studied as a model organism for regeneration and more recently, stem cell differentiation^23^. *H. vulgaris* is part of the *Hydra vulgaris* group, a group where species boundaries are uncertain and across which there is limited sequence diversity^32^. NCBI taxonomy considers *H. magnipapillata*^33^ and *H. attenuata* as heterotypic synonyms for *H. vulgaris* (NCBI:txid6087)^34^ and *H. vul*garis strain 105 specifically denotes the hydra previously described as *H. magnipapillata*. We use *H. vulgaris* as the current taxonomy but *H. magnipapillata* was used with the genome release^23^. The anthozoan *A. digitifera*, or stony coral, is one of the main species that comprises the architecture of coral reefs, sustaining some of the most diverse marine ecosystems on the planet^35^. Another anthozoan, the starlet sea anemone *N. vectensis*, has become a model organism for gene knockout studies due to its quick developmental cycle^36^. Lastly, the schyphozoan moon jellyfish *A. aurita* has long been of interest to the scientific community due to its unique body plan and complex life cycle, as well as periodic ‘blooms’ of jellyfish that can have destructive effects on marine ecosystems^37^. A broad study of the defensome of *N. vectensis* was conducted by Goldstone^21^, including searching the genome for putative CYPs. A total of 82 potential CYP genes were identified, but at that time they were not grouped into existing families or subfamilies due to low amino acid sequence similarity with other known CYPs (< 40%)^21^. However, phylogenetic investigation revealed that the majority of these genes belonged in cytochrome P450 clans 2 and 3, which include CYPs from other organisms that metabolize both exogenous and endogenous compounds. These data suggest that detailed genomic and phylogenetic analyses of cnidarian CYPs presents an exciting opportunity to identify novel CYP families and subfamilies given the early divergence of the cnidaria from the bilateral metazoan and the ecological diversity among individual cnidarian species. The available genome sequences provide the means for a broad scope analysis of cytochrome P450 genes in the cnidarian phylum as a whole, as well as a comparison between three major classes. The goal of this study was thus to identify and characterize the cytochrome P450 superfamily in cnidarians, which could lead to the discovery of novel CYP families with unique functional properties, illuminate the ways that these species respond to environmental stressors, and to provide insight into the evolution of CYP functional diversity within the Metazoa.

## Results

### Genome-wide annotation of cytochrome P450 genes

For each cnidarian genome, well curated CYPs from other species (human, fruit fly, zebrafish, nematode worm, and annelid worm) were used in the basic local alignment search tool (BLAST) to identify regions of the genome that contained a likely CYP gene. Annotations were individually curated to locate, where possible, start and stop codons, appropriate signal sequences for intron–exon boundaries, an appropriate size of approximately 500 amino acids in translated protein sequence, and the presence and location of the key CYP gene motifs (I-helix, K-helix, meander coil and heme loop); expressed sequence tag (EST) support and a match to the Pfam CYP hidden markov model (HMM) were used to confirm identified CYP genes. The 82 CYP regions previously identified in *N. vectensis* were curated without further searching of the genome. In total, there were 19 cytochrome P450 genes found in the *H. vulgaris* genome, 14 in the *A. digitifera* coral species, 22 in the *A. aurita* moon jelly, and 44 in *N. vectensis* that met all criteria during curation (start and stop codon, translated to 500 aa, contained all of the CYP motifs). Many of the *H. vulgaris* gene predictions had strong EST support and all predicted complete genes in all species matched the entirety of the Pfam CYP model (HMM) with high confidence. Exon number was low in many of the predicted cnidarian CYP genes, with typically only 1–3 exons per gene; the only cnidarian CYPs with greater than 5 exons were found in the coral *A. aurita*.

There were several ‘partial’ CYPs genes identified in each species that were missing several hundred amino acid residues at the N-terminus or C-terminus of the protein (thus missing start and/or stop codons), but still contained all four of the key CYP motifs. There were 5 ‘partial’ CYPs in *H. vulgaris*, 10 in the *A. digitifera* coral, 15 in the *A. aurita* moon jelly, and 25 in *N. vectensis*. These partial genes contained enough genetic information that they are presumed functional, even though they are not completely resolved in the current genome assemblies available for each species. A list of all the ‘complete’ and ‘partial’ genes from each species, complete with their genomic location and length is available in Table S1. Each of these was assigned a formal name by the CYP Nomenclature Committee.

The length of each sequence and conservation of signature CYP motifs are summarized in Table 1 (*H. vulgaris*), Table 2 (*A. digitifera*), and Table 3 (*A. aurita*) for each of the newly identified complete and partial genes. The K-helix and heme loop motifs were the most highly conserved in all three cnidarian species; the glutamic acid and arginine residues in the K-helix were present in every gene and the only deviation in the heme loop was a fairly conservative G > A substitution in the *A. aurita* CYP3552G1 and a deletion in the CYP20 proteins we have observed in other metazoans (and noted in^38^). The I-helix and meander coil had significantly more variation between different CYP genes; the I-helix is degenerate in the CYP20 genes (a feature we and others^38^ have observed in metazoan CYP20s; Lemaire personal communication and data not shown) and while the phenylalanine, proline and arginine residues in the meander coil are highly conserved, the others vary considerably. This type of variation in the I-helix and meander coil is not uncommon in CYPs from other species and thus is not surprising for the cnidarians.

**Table 1:** The occurrence and position of conserved cytochrome P450 motifs in the *H. vulgaris* CYPome.

**CYP**	Length	I-helix		K-helix		Meander Coil		Heme Loop	
		AA	[AG]G-x-[DE]T[TS]	AA	E-x-x-R	AA	FDPER	AA	F-x-x-G-x-R-x-C-x-G
CYP4LA1	491	302	E**G**H**DTT**	358	**E**SL**R**	410	**F**I**PER**	431	**F**SA**G**P**R**N**C**I**G**
CYP4LA2	502	300	**AG**H**DT**I	356	**E**SM**R**	408	**F**I**PER**	429	**F**SA**G**S**R**N**C**L**G**
CYP4LA3	497	308	E**G**H**DTT**	364	**E**SL**R**	416	**F**I**PER**	437	**F**SA**G**P**R**N**C**I**G**
CYP4LA4	498	309	E**G**H**D**S**T**	365	**E**SM**R**	417	**F**I**PER**	438	**F**SA**G**P**R**N**C**I**G**
CYP4LA5	505	316	E**G**H**DTT**	372	**E**SL**R**	424	**F**I**PER**	424	**F**SA**G**P**R**N**C**I**G**
CYP4LB1	506	291	**AG**H**DTT**	349	**E**SL**R**	401	**F**I**PER**	422	**F**SA**G**P**R**N**C**I**G**
CYP20A1	496	–	**–**	360	**E**VM**R**	412	**FDP**D**R**	433	**F**GFA**G**K**R**K**C**P**G**
CYP3352A1	497	297	**AG**S**ETS**	354	**E**TL**R**	407	**F**Y**PER**	430	**F**SS**G**P**R**S**C**I**G**
CYP3352A2	447	303	**AG**S**ETS**	360	**E**TL**R**	413	**F**Y**PER**	436	**F**SS**G**P**R**S**C**L**G**
CYP3352A3	493	298	**AG**S**ETS**	355	**E**TL**R**	408	**F**Y**PER**	431	**F**SN**G**P**R**S**C**L**G**
CYP3352A4	493	298	**AG**S**ETS**	355	**E**TL**R**	408	**F**Y**PER**	431	**F**SG**G**P**R**A**C**L**G**
CYP3352A5	326	131	**AG**S**ETS**	188	**E**TL**R**	241	**F**Y**PER**	264	**F**SS**G**P**R**S**C**L**G**
CYP3352A6	322	127	**AG**S**ETS**	184	**E**TL**R**	237	**F**Y**PER**	260	**F**SG**G**P**R**S**C**L**G**
CYP3352A7	308	129	**AG**S**ETS**	169	**E**TL**R**	222	**F**Y**PER**	245	**F**SG**G**P**R**S**C**L**G**
CYP3352B1	505	300	S**G**S**ETT**	357	**E**TL**R**	410	**F**N**P**Y**R**	433	**F**ST**G**L**R**A**C**L**G**
CYP3352C1	506	300	**AG**T**ET**A	357	**E**TL**R**	411	**F**N**P**Y**R**	434	**F**SA**G**T**R**V**C**L**G**
CYP3352C2	518	300	**AG**T**ET**A	357	**E**TL**R**	357	**F**N**P**Y**R**	434	**F**SA**G**T**R**V**C**L**G**
CYP3352D1	506	300	**GG**V**ETT**	357	**E**SL**R**	410	**F**N**P**Y**R**	433	**F**SI**G**L**R**A**C**L**G**
CYP3352D2	507	260	**AG**S**ETT**	317	**E**CL**R**	370	**F**N**P**H**R**	393	**F**SA**G**T**R**V**C**L**G**
CYP3352D3	499	276	T**G**S**ETT**	333	**E**CL**R**	386	**F**N**P**Y**R**	409	**F**SA**G**T**R**V**C**L**G**
CYP3352D4	475	276	T**G**S**ETT**	333	**E**CL**R**	386	**F**N**P**Y**R**	409	**F**SA**G**T**R**V**C**L**G**
CYP3352D5	444	300	**GG**S**ETT**	357	**E**CL**R**	410	**F**N**P**Y**R**	433	**F**SA**G**T**R**V**C**L**G**
CYP3353A1	489	295	**AG**S**ETT**	351	**E**TL**R**	404	**F**N**P**M**R**	427	**F**SA**G**P**R**G**C**I**G**
CYP3354A1	499	299	**AG**T**ETT**	355	**E**TL**R**	408	**FDP**K**R**	431	**F**SA**G**A**R**V**C**L**G**

‘Length’ indicates the total length of the translated protein sequence, and ‘AA’ is the position of the first amino acid residue for each motif. Bolded residues indicate residues that were conserved.

**Table 2:** The occurrence and position of conserved cytochrome P450 motifs in the *A. digitifera* CYPome.

**CYP**	Length	I-helix		K-helix		Meander Coil		Heme Loop	
		AA	[AG]G-x-[DE]T[TS]	AA	E-x-x-R	AA	FDPER	AA	F-x-x-G-x-R-x-C-x-G
CYP375B3	504	303	**AG**V**DTT**	360	**ETLR**	412	**F**K**PER**	439	**F**GF**G**T**R**M**C**L**G**
CYP3094B1	504	309	**AG**L**ETS**	366	**E**VL**R**	419	**F**N**PER**	442	**F**GA**G**R**R**G**C**L**G**
CYP3094H1	440	284	**AG**S**ET**L	342	**E**AL**R**	395	**FDP**N**R**	418	**F**SA**G**R**R**V**C**L**G**
CYP3094J1	485	290	**AG**L**ETT**	347	**E**VL**R**	400	**F**D**P**S**R**	423	**F**GA**G**R**R**V**C**L**G**
CYP3094K1	451	280	**AG**S**D**I**T**	337	**E**IM**R**	390	**FDP**T**R**	414	**F**GG**G**L**R**A**C**M**G**
CYP3094K2	451	280	**AG**S**D**I**T**	337	**E**IM**R**	390	**FDP**T**R**	414	**F**GG**G**L**R**A**C**M**G**
CYP3339A1	516	311	**AG**Y**ETS**	369	**E**AL**R**	421	**FDPER**	442	**F**GS**G**P**R**V**C**I**G**
CYP3339A2	462	267	**AG**Y**ETS**	326	**E**SQ**R**	378	**FDPER**	399	**F**GA**G**P**R**N**C**I**G**
CYP3339A3	511	317	**AG**Y**ETS**	376	**E**AL**R**	428	**FDPER**	449	**F**GA**G**P**R**N**C**I**G**
CYP3339A4	493	299	**AG**Y**ETS**	358	**E**TL**R**	410	**FDPER**	431	**F**GA**G**P**R**N**C**I**G**
CYP3342A5	228	36	**AG**Y**ETS**	94	**E**TL**R**	146	**FDPER**	167	**F**GH**G**PHN**C**V**G**
CYP3343A1	481	291	**GG**Y**E**KL	347	**E**IL**R**	400	**F**N**P**Y**R**	422	**F**ST**G**D**R**K**C**P**G**
CYP3344A1	489	291	**AG**I**DT**V	348	**E**TL**R**	401	**F**N**P**H**R**	424	**F**ST**G**G**R**R**C**L**G**
CYP3346A1	489	290	**AG**F**ETS**	347	**E**LL**R**	400	**F**N**P**R**R**	424	**F**SG**G**R**R**K**C**P**G**
CYP3346A2	476	276	**GG**AI**DTS**	334	**E**LL**R**	387	**F**N**P**R**R**	411	**F**SV**G**R**R**Q**C**P**G**
CYP3346A3	476	276	**GG**AI**DTS**	334	**E**LL**R**	387	**F**N**P**R**R**	411	**F**SV**G**R**R**Q**C**P**G**
CYP3346A4	492	293	**AG**F**DTS**	350	**E**LL**R**	403	**F**C**P**S**R**	427	**F**SA**G**R**R**K**CPG**
CYP3346A5	359	160	**AG**F**ETS**	217	**E**LL**R**	270	**F**N**P**K**R**	294	**F**SG**G**R**R**K**C**P**G**
CYP3346A6	491	313	**AG**F**ETS**	370	**E**LL**R**	423	**F**Y**P**R**R**	447	**F**SG**G**R**R**K**C**P**G**
CYP3348A1	397	206	**AG**Y**ETT**	262	**E**SL**R**	315	**F**N**P**Y**R**	337	**F**GA**G**R**R**V**C**A**G**
CYP3348A2	403	207	**AG**Y**ETT**	263	**E**SL**R**	316	**F**N**P**Y**R**	338	**F**GA**G**R**R**V**C**A**G**
CYP3348B6	346	197	**AG**Y**ETT**	254	**E**AL**R**	307	**F**N**P**Q**R**	329	**F**SA**G**R**R**V**C**A**G**
CYP3348B7	348	197	**AG**Y**ETT**	254	**E**AL**R**	307	**F**N**P**H**R**	329	**F**SA**G**R**R**V**C**A**G**
CYP3355C1	495	298	**AG**M**ETT**	355	**E**TL**R**	408	**F**R**P**D**R**	431	**F**SG**G**R**R**S**C**L**G**

‘Length’ indicates the total length of the translated protein sequence, and ‘AA’ is the position of the first amino acid residue for each motif. Bolded residues indicate residues that were conserved.

**Table 3:** The occurrence and position of conserved cytochrome P450 motifs in the *A. aurita* CYPome.

**CYP**	Length	I-helix		K-helix		Meander Coil		Heme Loop	
		AA	[AG]G-x-[DE]T[TS]	AA	E-x-x-R	AA	FDPER	AA	F-x-x-G-x-R-x-C-x-G
CYP4KY1	525	332	E**G**H**DTT**	390	**E**SL**R**	443	**F**I**PER**	464	**F**SA**G**P**R**N**C**V**G**
CYP20A1	481	–	**–**	348	**E**TL**R**	400	**FDPER**	423	**F-**A**G**K**R**K**C**P**G**
CYP375E1	440	248	**AG**V**DTT**	305	**E**TL**R**	357	**F**K**PER**	380	**F**GF**G**V**R**M**C**L**G**
CYP375E2	440	248	**AG**V**DTT**	305	**E**TL**R**	357	**F**K**PER**	380	**F**GF**G**V**R**M**C**L**G**
CYP3336A1	502	310	**AG**HH**TS**	367	**E**AM**R**	419	YN**P**D**R**	441	**F**AR**G**P**R**M**C**I**G**
CYP3336B1	504	309	**AG**H**ETT**	366	**E**SM**R**	418	**F**N**P**D**R**	442	**F**SR**G**P**R**M**C**I**G**
CYP3336B2	332	143	**AG**STSL	196	**E**SM**R**	248	**F**N**P**D**R**	270	**F**SR**G**P**R**M**C**I**G**
CYP3336B3	323	134	**AG**STSL	187	**E**SM**R**	239	**F**N**P**D**R**	261	**F**SR**G**P**R**M**C**I**G**
CYP3337A1	474	315	**AG**F**ETT**	372	**E**SL**R**	424	**FDPER**	447	**F**SN**G**P**R**N**C**I**G**
CYP3338A1	521	330	**A**AV**DTT**	387	**E**SM**R**	439	**F**K**P**K**R**	462	**F**GY**G**V**R**M**C**L**G**
CYP3341A1	501	309	**AG**Y**DTS**	366	**E**TL**R**	418	**F**K**PER**	439	**F**GA**G**P**R**N**C**I**G**
CYP3341A2	501	309	**AG**Y**DTS**	366	**E**TL**R**	418	**F**K**PER**	439	**F**GA**G**P**R**N**C**I**G**
CYP3341A3	502	309	**AG**Y**E**S**T**	366	**E**TL**R**	418	**F**K**PER**	439	**F**GM**G**P**R**N**C**I**G**
CYP3341A4	228	123	**AG**Y**DTS**	182	**E**TL**R**	228	**F**PDPE	255	**F**GM**G**P**R**N**C**I**G**
CYP3341A5	225	34	**AG**Y**DTS**	93	**E**TL**R**	139	**F**PDPE	166	**F**GM**G**P**R**N**C**I**G**
CYP3341A6	227	34	**AG**Y**E**S**T**	91	**E**TL**R**	143	**F**K**PER**	164	**F**GM**G**P**R**N**C**I**G**
CYP3341A7	226	34	**AG**Y**DTS**	91	**E**TL**R**	137	**F**PDPE	164	**F**GM**G**P**R**N**C**I**G**
CYP3341A8	226	34	V**G**Y**DTS**	91	**E**TL**R**	143	**F**K**PER**	164	**F**GM**G**P**R**N**C**I**G**
CYP3350A1	499	302	**AG**S**DTT**	358	**E**VL**R**	411	**F**Q**PER**	433	**F**SA**G**K**R**I**C**L**G**
CYP3350B1	498	305	T**G**T**ETT**	360	**E**VL**R**	413	**F**A**PER**	435	**F**SV**G**K**R**V**C**L**G**
CYP3350B2	220	27	T**G**T**ETT**	82	**E**VL**R**	135	**F**A**PER**	157	**F**SV**G**K**R**V**C**L**G**
CYP3351A1	316	122	**AG**V**DTT**	179	**E**TL**R**	231	**F**M**PER**	254	**F**SI**G**R**R**R**C**P**G**
CYP3351A2	469	275	**AG**V**DTT**	332	**E**TL**R**	384	**F**M**PER**	407	**F**SI**G**R**R**R**C**P**G**
CYP3352E1	499	302	**AG**S**ETT**	359	**E**TL**R**	412	**F**I**PER**	435	**F**SA**G**R**R**V**C**L**G**
CYP3352E2	499	302	**AG**S**ETT**	359	**E**TL**R**	412	**F**I**PER**	435	**F**SA**G**R**R**V**C**L**G**
CYP3352F1	495	302	**AG**A**ETT**	358	**E**TL**R**	411	**F**R**PER**	434	**F**GA**G**R**R**V**C**V**G**
CYP3352F2	495	302	G**G**A**ETT**	358	**E**TL**R**	411	**F**R**PER**	434	**F**GA**G**R**R**V**C**L**G**
CYP3352F3	487	291	**AG**S**ETT**	347	**E**IF**R**	399	**F**K**PER**	422	**F**GA**G**R**R**G**C**L**G**
CYP3352F4	503	302	S**G**A**ETT**	358	**E**TL**R**	411	**F**K**PER**	434	**F**GA**G**R**R**V**C**I**G**
CYP3352F5	328	127	S**G**A**ETT**	183	**E**TL**R**	236	**F**K**PER**	259	**F**GA**G**R**R**V**C**I**G**
CYP3552G1	506	300	**AG**S**ETT**	357	**E**VL**R**	410	**F**N**PER**	433	**F**SA**G**R**R**V**C**LA
CYP3352H1	498	303	**A**AI**DTT**	360	**E**TL**R**	413	**F**K**PER**	436	**F**SA**G**K**R**V**C**F**G**
CYP3352H2	322	127	**A**A**IETT**	184	**E**TA**R**	237	**F**Q**PER**	260	**F**SA**G**K**R**V**C**F**G**
CYP3352H3	498	303	**A**A**IETT**	360	**E**TA**R**	413	**F**Q**PER**	436	**F**SA**G**K**R**V**C**F**G**
CYP3352H4	498	303	**A**AT**ETT**	360	**E**SA**R**	413	**F**Q**PER**	436	**F**SA**G**K**R**V**C**F**G**
CYP3352H5	322	127	**A**AT**ETT**	184	**E**SA**R**	237	**F**Q**PER**	260	**F**SA**G**K**R**V**C**F**G**
CYP3352H6	498	303	**A**A**IETT**	360	**E**TA**R**	413	**F**Q**PER**	436	**F**SA**G**K**R**V**C**F**G**

‘Length’ indicates the total length of the translated protein sequence, and ‘AA’ is the position of the first amino acid residue for each motif. Bolded residues indicate residues that were conserved.

As with all genome annotations of CYPs, multiple ‘fragments’ of CYPs were identified; the fragments varied in length, were missing one or more of the main CYP motifs, and it was unclear on whether they represented functional genes or pseudogenes (Table S2): 11 fragment genes in *H. vulgaris*, 34 in *A. digitifera*, 31 in *A. aurita*, and 14 in *N. vectensis*. Based on the complete and partial CYP gene sequences and setting aside the fragmentary gene sequences, the cnidarian genomes contain a minimum of 24 (*H. vulgaris* and *A. digitifera*), 37 (*A. aurita*), or 69 (*N. vectensis*) functional CYP genes and a total of 24 novel CYP families and 12 new subfamilies. Two of the new families are exclusive to *H. vulgaris* (CYP3353, CYP3354), another two are exclusive to *A. digitifera* (CYP3344, CYP3346), six are exclusive to *N. vectensis* (CYP377, CYP443, CYP3340, CYP3345, CYP3347, CYP3349), and four are exclusive to *A. aurita* (CYP3336-3337, CYP3341, CYP3351). In total, more than 85% of the complete or partial genes identified are from new CYP families and, other than the CYP20 genes and one CYP16 in *N. vectensis*, the rest are from new subfamilies within the CYP3, CYP4 and CYP46 clans.

#### Phylogenetic analysis of cnidarian cytochrome P450 sequences

**Figure 2:**
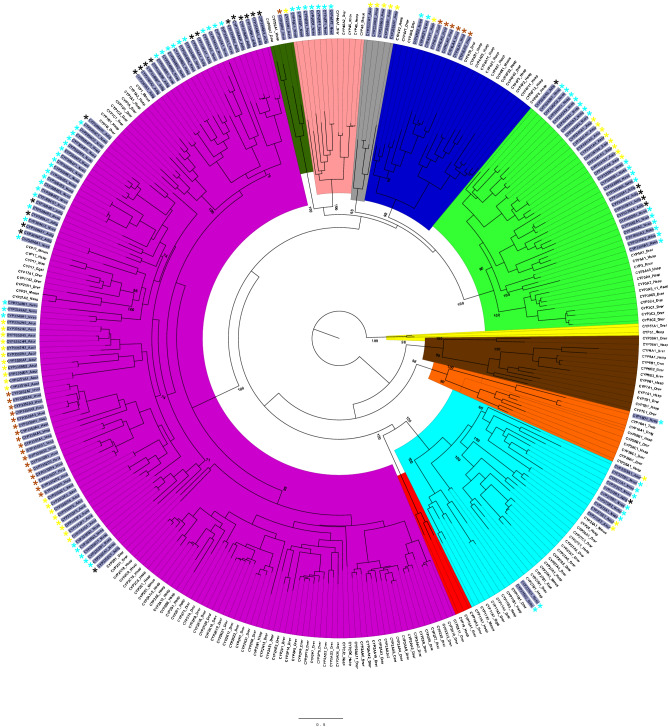
Maximum likelihood phylogenetic tree of cnidarian cytochrome P450 enzymes. The tree was generated with rapid bootstrapping and a gamma distribution with the JTT substitution matrix. Each CYP clan was assigned a different colour on the tree branches: clan 2 is purple, clan 3 is light green, clan 4 is blue, clan 7 is brown, clan 19 is red, clan 20 is dark green, clan 26 is orange, clan 46 is salmon, clan 51 is yellow, mitochondrial clan is turquoise. The five *A. aurita* CYPs that do not fit into established metazoan clans are light grey. The cnidarian CYPs are highlighted in dark grey on the perimeter of the tree. The different coloured asterisks indicate which cnidarian each CYP is from: *H. vulgaris* is brown, *A. digitifera* is black, *A. aurita* is yellow, *N. vectensis* is turquoise. The tree is rooted with the CYP51 family. Bootstrap values for each CYP clan, major CYP family divisions within each clan and major cnidarian clades are indicated on the tree. Bootstrap support for the placement of the branches that separate the individual clans was low (less than 50). Species acronyms are based on the family and species: Drer—zebrafish, Ggal—chicken, Hsap—human, Mmus—mouse, Rnor- rat, Xlae—frog, Trub—fugu pufferfish, Dnig—green pufferfish, Hvul—brown hydra, Adig—stony coral, Aaur—moon jellyfish, Nvec—starlet sea anemone.

The phylogenetic relationships between the cnidarian CYP genes are shown in Fig. 2. This maximum likelihood tree contains all of the complete and partial CYPs from each cnidarian species, but not the fragmentary genes. The phylogeny includes CYPs from humans, zebrafish and other vertebrates from eighteen vertebrate CYP families: CYP1-5, CYP7, CYP8, CYP11, CYP16, CYP17, CYP19-21, CYP24, CYP26, CYP27, CYP46 and CYP51. There are a total of 330 CYP sequences included in this tree. The vertebrate CYPs were clustered into their clans as expected and all clans had high bootstrap support (greater than 90%). Known species phylogeny was evident within the CYP gene families, both for the vertebrate species and those families where there were sequences from multiple cnidarian species. For example, *N. vectensis* and *A. digitifera* CYPs (the two anthozoan species) often clustered together, with representatives from each species in the CYP3094, CYP3339-CYP3340, CYP3342, and CYP3343-CYP3348 families clustered in the clan 2 and clan 3 clades. Likewise, *H. vulgaris* and *A. aurita* sequences always clustered together throughout the tree (CYP3351-3354 in clan 2, CYP4 and CYP20). In general, the cnidarian genes formed distinct clades within each clan (for example, the clan 2 *H. vulgaris*, clan 4 *H. vulgaris* and clan 3 *A. aurita* CYPs).

Clan 2 contained the largest number of cnidarian sequences, most of which were in clades separate from any vertebrate sequences. Nonetheless, there were cnidarian sequences from the CYP3094 and CYP3349 family that clustered close to the CYP17 and CYP21 families and several cnidarian families (CYP3346, CYP3347, CYP3343, and CYP3348) that clustered with the CYP1 family. Vertebrate CYP27 and CYP24 families clustered with the cnidarian CYP3338, CYP377, and CYP375 families in the mitochondrial clan except for the CYP375D subfamily in *N. vectensis*, which is basal to the same clade. Phylogenetic analysis also had clear support for cnidarian CYP4, CYP20 and CYP46 family members. Interestingly, the phylogenetic results also supported *N. vectensis* having several genes related to CYP3N (CYP3662A1-3) and CYP3P (CYP3662B1) subfamilies, in addition to novel cnidarian CYP families (notably CYP3340) that clustered close to the vertebrate CYP5s in clan 3.

**Figure 3:**
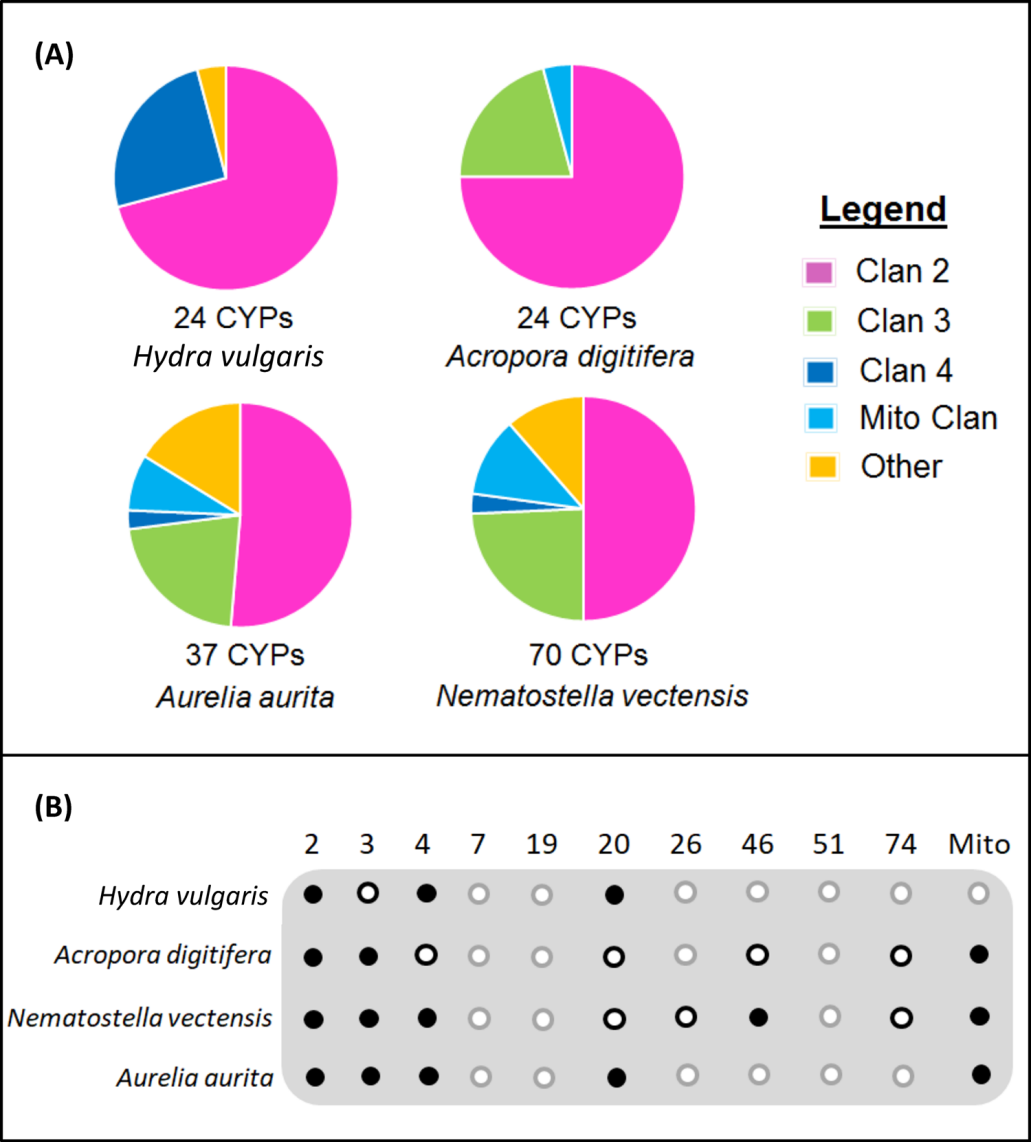
The distribution of cytochrome P450 genes among the metazoan CYP clans in four cnidarian species. (**A**) The proportion of each species’ CYPome that falls within the major metazoan clans (clan 2, clan 3, clan 4 and the mitochondrial clan). The number of CYP genes included in the analysis is indicated under each chart and includes all of the complete and partial CYPs from each species. Clan 16, clan 20 and clan 46 CYPs, as well as five *A. aurita* CYPs that did not fall discretely within any of the established metazoan CYP clans were designated as ‘Other’. (**B**) The presence or absence of each of the twelve metazoan CYP clans within each cnidarian species. Each column represents a different clan, and each row represents a different species. A filled black dot means that the clan was identified in the complete or partial genes, a white dot with a black outline means that the clan was identified in the fragmentary genes, and a white dot with a grey outline means that the clan was not found in the data. We have included CYP16 in Clan 16 because this clan designation has recently been determined by the nomenclature committee and other phylogenetic analyses support that CYP16 forms a separate clan^61^. In our phylogenetic analyses, the CYP16 genes clustered with Clan 26 genes (Fig. 2).

With the exception of five genes in *A. aurita* (CYP3336A1, CYP3336B1, CYP3336B2-1, CYP3336B2-2, and CYP3337A1), all of the cnidarian CYPs could be grouped into existing metazoan CYP clans. The proportion of each CYPome that fell into the major metazoan clans is shown in Fig. 3A. Clan 2 genes made up between 50 and 75% of each species’ CYPome, making it the most prevalent clan in each cnidarian genome. Interestingly, clan 3 was the second most prevalent (20–25%) for all of the cnidarians other than *H. vulgaris*, which lacked any complete or partial CYPs from this clan. While clan 4 CYPs represented a small portion of the *N. vectensis* and *A. aurita* CYPomes and were completely absent in *A. digitifera*, a relatively large proportion (25%) of the *H. vulgaris* CYPs fell in this clan. In total numbers this translates to only six clan 4 CYPs in *H. vulgaris* compared to two and one in *N. vectensis* and *A. aurita*, respectively. The mitochondrial clan CYPs were not highly prevalent in any of the cnidarian species (roughly 4–11% of the total CYPomes, except for *H. vulgaris,* where they were absent). Overall, when examining the presence/absence of the twelve metazoan CYP clans for each of the cnidarian species, the same clans were not found in all cnidarian genomes (Fig. 3B, Table S4). However, inclusion of fragmentary CYP genes finds clans 2, 3, 4 and 20 are present in all of four cnidarian species.

**Figure 4:**
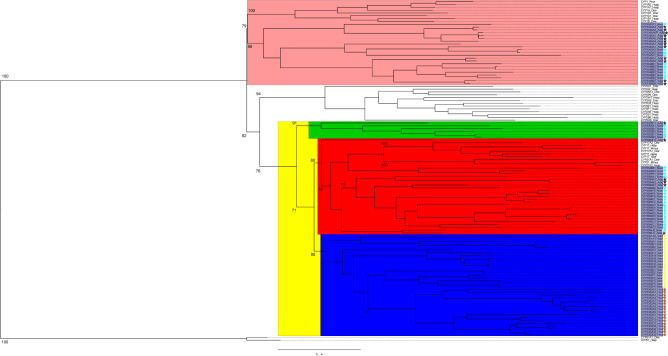
Maximum-likelihood phylogenetic tree of cnidarian clan 2 cytochrome P450 enzymes. The tree was generated with rapid bootstrapping and a gamma distribution with the JTT substitution matrix. The CYP1 family and a sister cnidarian clade are salmon. The yellow branch identifies a large group of cnidarian CYPs and the vertebrate CYP17 and CYP21 families; this branch is further divided based on evolutionary distance to the vertebrate CYPs (blue is almost all CYPs that clustered directly with the vertebrate sequences, while the red and green groups both clustered outside of the vertebrate sequences). The cnidarian CYP names are highlighted in dark grey on the tree. The different coloured asterisks indicate which cnidarian each CYP is from: *H. vulgaris* is brown, *A. digitifera* is black, *A. aurita* is yellow, *N. vectensis* is turquoise. The tree is rooted with the CYP51 family. Bootstrap values for major CYP family divisions and major cnidarian clades are indicated on the tree. Species acronyms are based on the family and species: Drer—zebrafish, Ggal—chicken, Hsap—human, Mmus—mouse, Rnor- rat, Xlae—frog, Hvul—brown hydra, Adig—stony coral, Aaur—moon jellyfish, Nvec—starlet sea anemone.

**Figure 5:**
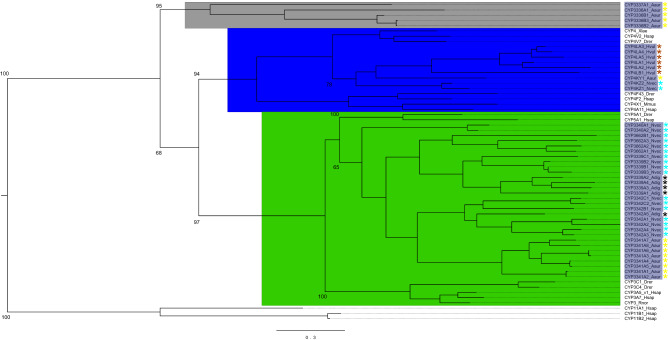
Maximum-likelihood phylogenetic tree of cnidarian clan 3 and clan 4 cytochrome P450 enzymes. The tree was generated with rapid bootstrapping and a gamma distribution with the JTT substitution matrix. The Clan 3 CYPs are light green, the Clan 4 CYPs are dark blue, and the CYPs that do not fall within a clan are grey. The cnidarian CYP names are highlighted in dark grey on the tree. The different coloured asterisks indicate which cnidarian each CYP is from: *H. vulgaris* is brown, *A. digitifera* is black, *A. aurita* is yellow, *N. vectensis* is turquoise. The tree is rooted with the CYP11 family. Bootstrap values for each CYP clan, major CYP family divisions within each clan and major cnidarian clades are indicated on the tree. Species acronyms are based on the family and species: Drer—zebrafish, Hsap—human, Mmus—mouse, Rnor- rat, Xlae—frog, Hvul—brown hydra, Adig—stony coral, Aaur—moon jellyfish, Nvec—starlet sea anemone.

To better examine CYP family associations, phylogenetic trees were generated with only clan 2 sequences, including the CYP1, CYP2, CYP17 and CYP21 vertebrate families (Fig. 4; 121 sequences) or only clan 3 and 4 sequences, including the CYP3, CYP4 and CYP5 vertebrate families (Fig. 5; 61 sequences). The five *A. aurita* CYPs that did not place within an existing metazoan clan in Fig. 2, but were basal to the clan 3 and 4 CYPs, were included in the clan 3 and 4 tree. These subtrees generally had higher bootstrap support than the main tree and it was clear that the cnidarian CYPs formed distinct groups within the larger clan 2 clade; this pattern was observed both on the large tree containing all of the cnidarian CYPs (Fig. 2) and within the clan 2-specific tree (Fig. 4), with strong bootstrap support separating the major groups in both cases. Within clan 2, the CYP3343-CYP3348 families in *N. vectensis* and *A. digitifera* formed a sister clade to the CYP1 family of enzymes, while all of the *H. vulgaris* and *A. aurita* CYPs in this clan, as well as the CYP3094, CYP3349 and CYP3355 families from *N. vectensis* and *A. digitifera,* formed a large clade with the CYP17 and CYP21 vertebrate families, with varying degrees of relatedness. The phylogenetic tree of the clan 3 and clan 4 CYPs verified that the five uncategorized *A. aurita* CYPs were basal to those clans (Fig. 5). The cnidarian clan 3 CYPs formed a clade of exclusively cnidarian genes that were most closely related to CYP5 genes. Similarly, all of the cnidarian CYP4 genes formed a distinct group within the clan 4 clade most closely related to CYP4V genes, and each of the genes belonged to new CYP4 subfamilies.

Each of the phylogenetic trees included annotated CYPs from vertebrate species. Inclusion of invertebrate CYPs from *D. melanogaster*, *C. elegans,* and *C. teleta* interfered negatively with tree construction, often resulting in low bootstrap support (< 50%). This has been reported in other studies using both vertebrate and invertebrate sequences^16,18^. Even with the vertebrate sequences, the deeper branches that separate the clans in Fig. 3 had some bootstraps under 50% (not shown on the tree), likely due to the evolutionary distance that separates the species between the various phyla. However, the relationship between the clans on this tree remains consistent with trends that are commonly seen in maximum-likelihood CYP analysis^12,13,21^ and bootstrap support for the individual clans remained high. The tree was rooted with the CYP51 family because CYP51 is the only CYP family found in all domains of life (including bacteria and viruses), and thus is thought to be the first eukaryotic CYP4^4,10^; CYP51 genes are commonly used to root trees in CYP phylogenetic analyses^19^. The same root was chosen for the clan 2 phylogeny and the mitochondrial clan CYP11 family was used to root the clan 3 and 4 phylogeny. Subtree roots were chosen from clans that typically form basal clades to the main clans being analyzed in those subtrees^12,13^.

## Discussion

### Cnidarian CYPomes

The genomic analysis of *H. vulgaris*, *N. vectensis*, *A. digitifera* and *A. aurita* demonstrated that cnidarian CYPomes are highly diverse, with 24 novel CYP families and 12 novel subfamilies involving more than 85% of the predicted cnidarian CYP genes. This means that a majority of cytochrome P450 genes in cnidarians are less than 40% similar to all other documented genes in this superfamily, a testament to more than 500 million years of natural selection and diversification^26^. There are other cnidarian gene families which have proven to be unique from their metazoan counterparts. For example, innexin transmembrane proteins identified in *H. vulgaris* were only 25% identical to similar genes in protostomes^39^. Similarly, while there are many identified precursors of neuropeptide signaling genes in *N. vectensis* (such as RPamides, RWamides, and VRamides), none of them are confirmed orthologs of bilaterian neuropeptides^40^. However, there is not always a significant difference between cnidarian sequences and other studied metazoans. The Wnt signaling protein family is largely conserved between cnidarians and vertebrates, as most major subfamilies are present in both phyla^41,42^. Additionally, an examination of the SOX transcription factor gene family in cnidarians identified clear bilaterian orthologs along with expected expression patterns^43^. This makes it clear that certain gene families arose in an early metazoan ancestor and remained largely unchanged in cnidarians, while others such as the cytochrome P450 superfamily arose early in the history of metazoans but were subject to significant change in this phylum. Using sequence similarity to known genes does have its challenges, particularly when there is bias in the taxonomic sampling across the tree of life. Much of the known CYP genes are from eukaryotes and of the metazoan sequences, vertebrates and insect sequences dominate (see the Cytochrome P450 Homepage; https://drnelson.uthsc.edu/CytochromeP450.html). Thus, it will be highly interesting to reassess the unique cnidarian genes and determine which are the result of duplications within the cnidarian lineage and which have non-vertebrate orthologs. Much better taxonomic sampling, both within and near the cnidarians, would be helpful to address this question.

There is clear diversity in the cytochrome P450 complement across the cnidarians as a phylum. Figure 3 demonstrates that, when considering gene number, proportion of CYPs belonging to particular clans, and the presence/absence of different CYP clans, none of the four species considered were exactly the same. Differences between the *N. vectensis* and *H. vulgaris* genome have been well documented, since they were the first two cnidarian species to be fully sequenced; both in terms of general observations such as genome size, but also in terms of other specific genes such as the 301 family epitheliopeptides and the SWT domain of receptors and secreted proteins^44^. Publication of the *A. digitifera* genome additionally identified that there are marked differences in the repertoire of TIR-domain-containing proteins between *A. digitifera*, *N. vectensis,* and *H. vulgaris*^24^. The four cnidarian species analyzed here differ in total CYP number and in the families and clans of CYPs that are present in their genomes. There are certain families that were exclusive to a particular species (Table S3); based on the phylogenetic history of the phylum^25,30^ (Fig. 1B) it is possible to infer when these families arose relative to the speciation events that formed the different classes and species. Table S3 identifies the CYP families by species and indicates likely gene gain or loss by species or class. In general, *N. vectensis* has a much larger CYP gene complement than the other cnidarians and that is largely based on gene gains, even in CYP families shared with *A. digitifera* (e.g. 17 CYP3094 genes versus 5 in *A.digitifera*; 7 CYP3342 genes versus 1 in *A. digitifera*). Gene expansions are common among CYP families in other species, including those as diverse as mouse, sea urchin, and mosquito. These expansions are often in CYP families involved in responding to the chemical environment, although in many cases little is known about the enzyme function. Gene expansions have been previously identified in Anthozoans^22^; what novel functions are afforded *N. vectensis* from the CYP gene expansions are not clear.

Fourteen of the new families (CYP377, CYP443, CYP3094, CYP3339, CYP3340, CYP3342-3349 and CYP3355) were exclusive to the anthozoan class, meaning these likely arose after the anthozoans diverged from the remainder of the cnidarians. Of these families, CYP3094, CYP3339, CYP3342, CYP3343, CYP3348 and CYP3355 are found in both *A. digitifera* and *N. vectensis,* indicating that these families likely arose after the anthozoan split from the rest of the cnidarians, but before the split between the two species within this class. CYP3344, CYP3346, and CYP3348 are exclusive to *A. digitifera* while CYP377, CYP3340, CYP3345, CYP3347, and CYP3349 are exclusive to *N. vectensis*, which means these families likely arose separately after the split of the lineages including these species. CYP443 was exclusive to *N. vectensis* in this gene set, but CYP443 genes have been found in *A digitifera, A. palmate, A. millepora, Aiptasia pallida* and *Anemonia viridis* (D. Nelson, unpublished data), suggesting this is gene family is not exclusive. CYP443 are in Clan74 and are found in only a few animal species. Five CYP families (CYP334, CYP3336-3338 and CYP3351) are exclusive to the scyphozoan species and just two (CYP3353 and CYP3354) are exclusive to the hydrozoan species. The CYP3352 family is present in both *H. vulgaris* and *A. aurita*, which means it likely arose after the anthozoan split, but before the split between hydrozoan and scyphozoans. The presence of multiple species-exclusive CYP families is likely the result of exposure to different selective pressures and environments over time. Even the two anthozoan species, which are presumed to be the most similar to each other based on phylogenetics, diverged roughly 520–490 million years ago^24^. That means there was a significant period of time (> 500 million years) when these cnidarian lineages were exposed to different environments, had access to different resources, and were differentially exposed to other factors that would influence CYP evolution.

### Cnidarian CYP Clans

The CYP clan framework was implemented as a means to describe the recurring deep branching clusters observed in metazoan CYP phylogenies^9^. The clans provide a way to describe and characterize the complexity of CYP evolution between species. Multiple phylogenetic analyses have established certain relationships between the metazoan CYP clans; for example, clan 3 and clan 4 consistently form sister clades in metazoan phylogenies^17,19,21^. These same patterns are observed in the trees generated for this study, although the bootstrap support is not high for this grouping in this phylogeny (Fig. 2). The presence and absence of each clan has been examined in multiple phyla, establishing expectations for which clans are present in different types of species. Almost all metazoans, excluding certain early-branching phyla such as ctenophores, have CYPs from clans 2, 3, 4, and the mitochondrial clan^19^. When fragmentary genes are considered, this is true for all of the cnidarian species analyzed (Fig. 3B). Large clan losses have been documented in certain phyla as well, particularly in nematodes and arthropods^19^. Our analysis indicates that clans 7 and 51 were lost in the cnidarian phylum as a whole; CYP19 is not present in any cnidarian genome (Fig. 3B). Examining the phylogenetic relationships between the cnidarian CYPs and clans provides conservative hypotheses about what types of CYPs are present in these species, based on the evolutionary distance between the sequences. Clan 2 genes were especially abundant in the cnidarian CYPomes and are discussed in detail, as is the particularly interesting loss of clan 51.

### Clan 2

In general, the clan 2 genes are the most prevalent CYPs in all four cnidarians (approximately 50% of the *N. vectensis* and *A. aurita* CYPomes and approximately 75% of the *A. digitifera* and *H. vulgaris* CYPomes), which is also the case in humans^7^, zebrafish^12^, the tunicate *Ciona intestinalis*^14^, the marine annelid *Capitella teleta*^18^, and the echinoderm *Stronglyocentrotus purpuratus*^15^. The clan 2 phylogenetic tree (Fig. 4) elaborates on the relationships seen in the larger phylogeny (Fig. 2), with high bootstrap support. None of the cnidarian genes clustered with the CYP2 family, which is prominent in vertebrates and includes enzymes that catalyze arachidonate and drug metabolism. In contrast, CYP1 genes and other CYP families (sometimes referred to as “CYP1-like”) have frequently been found to closely cluster with CYP1s in other species, such as tunicates^14^, annelids^18^, and the sea urchin^15^. In cnidarians, the CYP3343-CYP3348 families from *N. vectensis* and *A. digitifera*, formed a large clade with the CYP1 family, making these cnidarian genes strong candidates for xenobiotic metabolism. The CYP1 family is critical for the metabolism of planar, halogenated compounds such as polycyclic aromatic hydrocarbons in vertebrates^6,7^ and there is evidence that cnidarians (*N. vectensis* in particular) respond to PAHs and weathered oil through increased expression of certain CYPs as well as antioxidant enzymes such as superoxide dismutases and catalases^45,46^ (Berger and Tarrant personal communication).

A large group of cnidarian clan 2 CYPs form a clade with the CYP17 and CYP21 families, which are directly linked to steroidogenesis in vertebrates. The CYP17A1 gene in humans, also known as steroid 17α-monooxygenase/17,20-lyase, oxidizes pregnenolone or progesterone, which is one of the first steps in androgen or corticoid synthesis following cholesterol side-chain cleavage^47^. The CYP21A1 gene, or steroid 21-hydroxylase, catalyzes the hydroxylation of progesterone and 17α-hydroxyprogesterone, one of the intermediate steps in the synthesis of the stress hormone cortisol^48^. The CYP3094 and CYP3349 families in *N. vectensis* and *A. digitifera* form a clade immediately basal to the CYP17/21 genes, while the CYP3355 families were clearly outside of this clade (Fig. 4). There are a total of sixty-seven cnidarian enzymes in this large group of CYPs (25 N*. vectensis*, 6 *A. digitifera*, 17 *H*. *vulgaris* and 19 *Aurelia*), with particularly large expansions of the CYP3352 family in *H. vulgaris* and *A. aurita* and the CYP3094 family in *N. vectensis* and *A. digitifera*. Most species typically have a fairly low copy number of steroidogenic CYPs (for example, humans have only one CYP17A1 gene^7^ and zebrafish have only two^12^), which means it is unlikely that all of these genes are steroidogenic. Similarly, the presence of multiple clan 46 genes in *N. vectensis* is surprising. The CYP46 family, or cholesterol 24-hydroxylase, modifies cholesterol so that it can pass through the blood–brain barrier in humans and be metabolized in the liver^49^. In vertebrates these genes are usually present in low copy number, with only one in humans^7^ and two in zebrafish^12^. CYP46 copy number is likewise low in the scallop *Chlamys farreri* (2)^50^, the polychaete *C. teleta* (1)^18^, and the ctenophore *Mnemiopsis leidyi*^19^ but was much higher in four species of *Brachionus* rotifers (7–8 per species^51^). There are seven CYP46 genes in *N. vectensis*. Although the *N. vectensis* nervous system lacks a brain-like centre or core, the molecular function of these CYPs is presumed to be similar to their vertebrate homologs based on high sequence similarity^52^.

The capacity to synthesize estrogen de novo by cnidarians has been demonstrated in the anthozoans *Scleractinia*^53^ and *Montipora*^54^. In vertebrates, select CYP families (CYP11, CYP17, CYP19, CYP21, CYP51) and hydroxysteroid-dehydrogenase enzymes play important roles in steroidogenesis, including estrogen production^55^. However, there were few cnidarian CYPs that qualify as candidate homologs of vertebrate steroid synthesis genes. No cnidarian CYP gene clustered with the CYP19 or CYP51 families and the cnidarian CYPs in the mitochondrial clan clearly do not cluster with the CYP11 family (Fig. 2). Thus, if CYPs play a major role in de novo estrogen production in cnidarians, it is likely that the CYP family (or families) involved are phylogenetically distinct from the vertebrate genes, and pinpointing the exact genes will be challenging. The best candidates in our results are the CYPs in phylogenetic proximity to the steroidogenic CYP17 and CYP21 vertebrate families (Fig. 2), such as CYP3094K1, CYP3094K2, and CYP3094A1 from the anthozoans *N. vectensis* and *A. digitifera*.

### Clan 51

Clan 51, which consists entirely of the CYP51 family, was not present in any of the cnidarian genomes analyzed in this study, even when potential pseudogenes or gene fragments were considered. The lack of CYP51 genes is interesting as this family encodes lanosterol 14 α-demethylase, which catalyzes an essential step in the biosynthesis of cholesterol^56^. This family has been found in molluscs, annelids, and sponges in addition to vertebrates, but is absent in insects, crustaceans, and nematodes^19^, and some tunicates^57^. The absence of this family from the cnidarian species studied here implies that these species lack de novo cholesterol synthesis and would need to receive this important signaling molecule from their environment and diet. Studies on the evolution of the cholesterol biosynthesis genes found losses in Cnidaria^58^. This is in line with research that indicated cholesterol as the most prominent sterol (50–63%) in lipid samples of cnidarians as a result of their generally carnivorous diet^59^. There is varying data regarding de novo cholesterol biosynthesis in cnidaria^57^, as for example, some, but not all, anthozoa tested in one study exhibited de novo sterol synthesis^60^. Thus obligate dietary cholesterol may be a character that varies within cnidaria, and indeed even within anthozoa.

### Hydra vulgaris

The CYPome of *H. vulgaris* is of special interest due to several unique differences from the CYPomes of other species. There were 24 complete CYP genes predicted in *H. vulgaris* and only 11 fragments that may or may not be functional. This is smaller than most reported metazoan CYPome; to date, the sponge *Amphimedon queenslandica* with 35 genes^19^ and the tomato russet mite *Aculops lycopersici* with 23 genes^61^ have the smallest CYPomes. The results for *H. vulgaris* are a large departure from the 69 predicted CYPs in *N. vectensis*, which is comparable to most vertebrate CYPomes (usually around 50–100 genes)^19^ and other species such as the crustacean *Daphnia pulex* (75 CYPs)^17^ and the sea urchin *Stronglyocentrotus purpuratus* (120 CYPs)^15^. While there were only 24 CYPs predicted in *A. digitifera*, there were 34 fragments, which means it is possible that this coral CYPome is considerably larger.

Certain CYP families (CYP375, CYP3350) are present in the anthozoan and scyphozoan cnidarians, but absent in the hydrozoan *H. vulgaris*. The phylogenetic history of the cnidaria (Fig. 1B) suggests that any gene families present in anthozoans and scyphozoans arose in the ancestral cnidarian and would be present in hydrozoans as well unless lost. Thus, clan 3 genes were identified in all of the cnidarian species except for *H. vulgaris*, yet clan 3 CYPs are believed to have been present in very early animals, before the origins of cnidarians^19^. As indicated in Fig. 3B, a single gene fragment in *H. vulgaris* had high sequence similarity to CYP3 genes from other species, but it was missing the critical CYP heme loop motif (Table S2) and may represent a pseudogene. Regardless, it is clear that clan 3 genes have undergone a significant reduction in *H. vulgaris* when compared to the other cnidarians (Fig. 3A). A similar reduction in gene count is observed in the mitochondrial CYP clan in *H. vulgaris* (Fig. 3B).

All of this evidence suggests that extensive loss of CYP genes and clans has occurred in *H. vulgaris*, more so than the other cnidarian species analyzed, in agreement with observations by other researchers that considerable gene loss has occurred in the *Hydra* genome as a whole. For example, while *N. vectensis* has roughly 140 homeobox genes from nearly 60 gene families, *H. vulgaris* only has about 50 homeobox genes from 30 gene families (no such reduction has been observed in *A. digitifera*)^62^. The genomic origin of the Hox gene cluster is thought to be a close neighbour to the original cytochrome P450 locus that expanded to form the large CYP superfamily, which provides further support for the smaller *Hydra* CYPome^19^. More than half of the *H. vulgaris* genome is composed of repetitive elements (including transposable elements) and estimates of protein-coding gene number are significantly lower in this species^23^. There is no clear explanation for this reduction in genome size and loss of multiple gene families in *H. vulgaris*, although it is correlated with lack of a larval stage in the *H. vulgaris* life cycle. Given the reduced *H. vulgaris* CYPome, determining the function of the novel *H. vulgaris* CYPs is of particular interest.

### Annotation and phylogenetic analysis of cytochrome P450 genes

Precise gene annotation and phylogenetic analysis can be difficult when studying species that are distantly related to those with well-studied and defined CYPomes. The CYPs used to search the cnidarian genomes were well curated genes from humans, *D. rerio, D. melanogaster, C. elegans,* and *C. teleta*. As Tables 1, 2 and 3 indicate, all four significant P450 motifs are generally well conserved in the cnidarian CYPs. The I-helix motif ([AG]G-x-[DE]T[TS]) has been shown to play a role in proton delivery for the reactions that CYPs catalyze, while the K-helix (E-x-x-R) and meander coil (FDPER) are thought to stabilize the core structure of the protein through salt bridge interactions^63^. The heme loop (F-x-x-G-x-R-x-C-x-G) is arguably the most important of the four because it facilitates association with the heme cofactor in the active site^64^. Other than a few known exceptions (for example, the CYP20 family is missing the I-helix motif and contain a deletion in the heme loop (K. Pankov, personal observation)—both observed in our cnidarian data), the large majority of metazoan cytochrome P450 enzymes maintained the presence of these four motifs and their relative position in CYP proteins was conserved. Of the predicted cnidarian CYPs, there were only a few predicted amino acid substitutions in the I-helix or meander coil and perfect matches to the K-helix and heme loop motifs in all cases, suggesting accurate prediction of gene models in these cnidarian genomes. However, multiple partial genes and gene fragments were identified in each annotated genome, which could be the result of evolutionary processes or artifacts of genome sequencing and assembly (albeit 90% of the *H. vulgaris* and *A. digitifera* genomes are estimated to be present in the current assemblies)^23,24^. By our definition, partial sequences had sufficient length to identify all conserved CYP motifs, with a strong match to the P450 HMM, and EST coverage when applicable; increasing our confidence that the partial genes encode functional CYP genes but that their sequences include genome assembly gaps. For example, certain partial CYPs on Contig36189 for *H. vulgaris* and Contig NW_015441134.1 for *A. digitifera* have sequencing gaps directly upstream of the predicted genes. The predicted fragmentary genes are more problematic—additional research will be needed to determine if they reflect real pseudogenes or genome sequencing artifacts. However, pseudogenes are common in the CYP superfamily, since much of the diversity of cytochrome P450s arose from multiple tandem gene duplications^65^, and there is clear evidence for tandem CYP gene duplication in all of the cnidarian genomes (Table S1). There were 13 potential pseudogenes in the annelid *C. teleta* CYPome^18^ and 17 in the vertebrate *Fugu* CYPome^13^, comparable to the fragmentary genes found in the cnidarian genomes: 11 gene fragments in *H. vulgaris*, 34 in *A. digitifera*, 31 in *A. aurita,* and 14 in *N. vectensis*.

### Conclusion

Gene annotation has made it clear that there is significant diversity between cnidarian CYPomes, even when those species belong in the same class. Over 500 million years of evolutionary history has expanded cnidarian CYPomes in some clans (especially clan 2) and resulted in extensive loss in others (clan 3 and mitochondrial clan CYPs). Certain cnidarians have a relatively large and diverse set of CYPs (the starlet sea anemone and the moon jellyfish), while others have a smaller, less diverse CYP complement (the stony coral and the brown hydra). Overall, cnidarian CYPs are found in nine of the twelve metazoan CYP clans; clan 16 is the most recent^61^. Yet, our analysis identified 24 novel cnidarian CYP families. This presents exciting opportunities for discovery of new functional capabilities of the cytochrome P450 superfamily in the context of metazoan and cnidarian evolution. This study presents several cnidarian candidates for genes with detoxification roles (CYP3343-3348 in the anemone and coral) and many more from all four cnidarians that could be involved in steroidogenesis^66,67^. All of the predicted functional cytochrome P450s in *H. vulgaris*, *A. digitifera*, *N. vectensis,* and *A. aurita* have been identified with confidence. Functional assays and in silico methods such as structural modeling and substrate docking may provide additional clues to the role of these cnidarian enzymes.

## Methods

### Genome-wide annotation of cytochrome P450 genes

The first versions of the genome assemblies for each species (*H. vulgaris*, *A. digitifera*, *A. aurita*) were searched for cytochrome P450 genes. The Basic Local Alignment Search Tool (v2.2.31)^68^ was used to identify local alignments between the cnidarian genomes and a query that consisted of all annotated CYPs in humans and zebrafish (vertebrate) and select CYPs in *D. melanogaster* (arthropod), *C. elegans* (nematode), and *C. teleta* (annelid) in a TBLASTN search (protein query against a genome nucleotide). Though previous efforts had been successful with just vertebrate queries^18^, a large variety of sequences from across metazoans were included to maximize CYP gene identification. Only those BLAST high scoring pairs with expectation values of 1.0 × 10^–11^ or smaller were considered significant.

The JBrowse genome viewer (v1.12.1)^69^ was used to manually annotate the significant regions of each genome from the BLAST results, identifying start (ATG) and stop (TGA/TAA/TAG) codons, plus introns, exons, and splice site signals (GT/AG) at intron–exon boundaries. Expressed sequence tag (EST) data sets publicly available for both *H. vulgaris* and *A. aurita* were aligned to the respective genomes with BLAT (BLAST-like alignment tool)^70^ as an aid to identification of genes, intron–exon boundaries, and confirmation of gene expression. The *H. vulgaris* EST data set consisted of approximately 18,000 individual reads^23^ while the *A. aurita* EST data set contained only 77 reads^37^.

Potential CYPs in each cnidarian species were identified, considered full length at ~ 500 amino acid residues, and were matched to the well-curated cytochrome P450 HMM in the Pfam protein family database^71^ to confirm identity. The ScanProsite tool^72^ was used to verify the presence of four largely conserved CYP motifs: the I-helix, K-helix, meander coil and heme loop. Pfam and Prosite scans included the 82 N*. vectensis* CYPs previously identified but not named^21^. Each putative CYP was classified as complete (proper length with start and stop codon, all motifs present, and match to the HMM) or partial (presence of at least the entire ~ 120 amino acid region that contains all motifs, but clearly less than full length). Any potential CYP that was missing at least one of the Prosite motifs was considered a gene fragment. The resulting complete cnidarian CYPs were used as queries for another BLAST search of each species’ genome to ensure that all paralogs were identified.

### Phylogenetic analysis of cnidarian cytochrome P450 sequences

All of the complete and partial CYPs were included in phylogenetic analyses, plus the previously predicted CYP protein sequences for *N. vectensis*^21^, with all predicted CYP proteins assigned a formal name by the CYP Nomenclature Committee. Clustal Omega (v1.2.4)^73^ was used to generate a global multiple sequence alignment of all *H. vulgaris*, *A. digitifera*, *N. vectensis* and *A. aurita* sequences plus a variety of vertebrate CYPs, including all major families from humans and *Danio rerio*, and select families from *Mus musculus*, *Xenopus laevis*, *Rattus norvegicus*, *Gallus gallus*, *Takifugu rubripes*, and *Dichotomyctere nigroviridis* (over 300 sequences in total). Mesquite (v3.10)^74^ was utilized to remove poorly aligned regions of uncertain homology, especially at the termini of the protein sequences where significant variation is typically observed. The final trimmed alignment was used as input for the Randomized Axelerated Maximum Likelihood Program (RAxML v8.2.9)^75^, with the rapid generation algorithm (-x), a gamma distribution for among-site rate variation, the JTT substitution matrix with empirical amino acid frequencies, and 100 bootstrap replicates for assessment of phylogenetic confidence. The final maximum likelihood phylogenetic tree was visualized with Figtree (v1.4.3)^76^ and rooted using the CYP51 family of enzymes. Clan-specific multiple sequence alignments and phylogenetic trees were generated using the same process, restricted to include only sequences that clustered with a particular clan in the global phylogeny. The clan 2 tree was rooted with CYP51, while the clan 3 and 4 trees were rooted with the CYP11 family.

## Supplementary Information

Supplementary Information 1.

Supplementary Information 2.

Supplementary Information 3.

Supplementary Information 4.

Supplementary Information 5.

## Data Availability

No new sequence data were generated, previously published accessions are included in the manuscript.

## References

[1] Danielson PB (2002). The cytochrome P450 superfamily: Biochemistry, evolution and drug metabolism in humans. Curr. Drug Metab..

[2] Hill HAO, Roder A, Williams RJP (1970). Cytochrome P-450: Suggestions as to the structure and mechanism of action. Nature.

[3] Zhang LH, Rodriguez H, Ohno S, Miller WL (1995). Serine phosphorylation of human P450c17 increases 17,20-lyase activity: Implications for adrenarche and the polycystic ovary syndrome. Proc. Natl. Acad. Sci. USA.

[4] Lepesheva GI, Waterman MR (2007). Sterol 14α-Demethylase Cytochrome P450 (CYP51), a P450 in all biological kingdoms. Biochim. Biophys. Acta.

[5] Arnold C, Konkel A, Fischer R, Schunck WH (2010). Cytochrome P450-dependent metabolism of omega-6 and omega-3 long-chain polyunsaturated fatty acids. Pharmacol. Rep..

[6] Lewis DF (2003). Human cytochromes P450 associated with the phase 1 metabolism of drugs and other xenobiotics: A compilation of substrates and inhibitors of the CYP1, CYP2 and CYP3 families. Curr. Med. Chem..

[7] Lewis DF (2004). 57 varieties: The human cytochromes P450. Pharmacogenomics.

[8] Nelson DR, Koymans L, Kamataki T, Stegeman JJ, Feyereisen R, Waxman MR, Gotoh O, Coon MJ, Estabrook RW, Gunsalus IC, Nebert DW (1996). P450 superfamily: Update on new sequences, gene mapping, accession numbers and nomenclature. Pharmacogenet.

[9] Nelson DR (1998). Metazoan cytochrome P450 evolution. Comp. Biochem. Physiol. C Pharmacol. Toxicol. Endocrinol..

[10] Nelson DR (1999). Cytochrome P450 and the individuality of species. Arch. Biochem. Biophys..

[11] Nelson DR (2011). Progress in tracing the evolutionary paths of cytochrome p450. Biochem. Biophys. Acta.

[12] Goldstone JV, McArthur AG, Kubota A, Zanette J, Parente T, Jonsson ME, Nelson DR, Stegeman JJ (2010). Identification and developmental expression of the full complement of cytochrome P450 genes in zebrafish. BMC Genomics.

[13] Nelson DR (2003). Comparison of P450s from human and fugu: 420 million years of vertebrate P450 evolution. Arch. Biochem. Biophys..

[14] Goldstone JV, Goldstone HM, Morrison AM, Tarrant A, Kern SE, Woodin BR, Stegeman JJ (2007). Cytochrome P450 1 genes in early deuterostomes (tunicates and sea urchins) and vertebrates (chicken and frog): origin and diversification of the CYP1 gene family. Mol. Biol. Evol..

[15] Goldstone JV, Hamdoun A, Cole BJ, Howard-Ashby M, Nebert DW, Scally M, Dean M, Epel D, Hahn ME, Stegeman JJ (2006). The chemical defensome: environmental sensing and response genes in the *Stronglyocentrotus purpuratus* genome. Dev. Biol..

[16] Tijet N, Helvig C, Feyereisen R (2001). The cytochrome P450 gene superfamily in *Drosophila melanogaster*: annotation, intron-exon organization and phylogeny. Gene.

[17] Baldwin WS, Marko PB, Nelson DR (2009). The cytochrome P450 (CYP) gene superfamily in *Daphnia pulex*. BMC Genomics.

[18] Dejong CA, Wilson JY (2014). The cytochrome P450 superfamily complement (CYPome) in the annelid *Capitella teleta*. PLoS ONE.

[19] Nelson DR, Goldstone JV, Stegeman JJ (2013). The cytochrome P450 genesis locus: The origin and evolution of animal cytochrome P450s. Philos. Trans. R. Soc. B Biol. Sci..

[20] Guengerich FP, Cheng Q (2011). Orphans in the human cytochrome p450 superfamily: Approaches to discovering functions and relevance in pharmacology. Pharmacol. Rev..

[21] Goldstone JV (2008). Environmental sensing and response genes in cnidaria: The chemical defensome in the sea anemone *Nematostella vectensis*. Cell Biol Toxicol.

[22] Gold DA, Katsuki T, Yang L, Yan X, Regulski M, Ibberson D, Holstein T, Steele RE, Jacobs DK, Greenspan RJ (2019). The genome of the jellyfish *Aurelia* and the evolution of animal complexity. Nat. Ecol. Evolut..

[23] Chapman JA, Kirkness EF, Simakov O, Hampson SE, Mitros T, Weinmaier T, Rattei T, Balasubramanian PG, Borman J, Busam D (2010). The dynamic genome of *Hydra*. Nature.

[24] Shinzato C, Shoguchi E, Kawashima T, Hamada M, Hisata K, Tanaka M, Fujie M, Fujiwara M, Koyanagi R, Ikuta T (2011). Using the *Acropora digitifera* genome to understand coral responses to environmental change. Nature.

[25] Brusca RC, Wendy M, Shuster SM (2016). Invertebrates.

[26] Sullivan JC, Retizel AM, Finnerty JR (2006). A high percentage of introns in human genes were present early in animal evolution: Evidence from the basal metazoan *Nematostella vectensis*. Genome Inform..

[27] Dohrmann M, Wörheide G (2017). Dating early animal evolution using phylogenomic data. Sci. Rep..

[28] Gold DA (2018). Life in changing fluids: A critical appraisal of swimming animals before the Cambrian. Integr. Comp. Biol..

[29] Park E, Hwang DS, Lee JS, Song JI, Seo TK, Won YJ (2012). Estimation of divergence times in cnidarian evolution based on mitochondrial protein-coding genes and the fossil record. Mol. Phylogenet. Evol..

[30] Kayal E, Bentlage B, Pankey MS, Ohdera AH, Medina M, Plachetzki DC, Collins AG, Ryan JF (2018). Phylogenomics provides a robust topology of the major cnidarian lineages and insights on the origins of key organismal traits. BMC Evol. Biol..

[31] Leclère L, Horin C, Chevalier S, Lapébie P, Dru P, Peron S (2019). The genome of the jellyfish *Clytia hemisphaeric* and the evolution of the cnidarian life-cycle. Nat. Ecol. Evol..

[32] Martínez DE, Iñiguez AR, Percell KM, Willner JB, Signorovitch J, Campbell RD (2010). Phylogeny and biogeography of *Hydra* (Cnidaria: Hydridae) using mitochondrial and nuclear DNA sequences. Mol. Phylogenet. Evol..

[33] Itô T (1947). A new fresh-water polyp, *Hydra magnipapillata*, n. sp. from Japan. Sci. Rep..

[34] Pallas, P. S. Elenchus Zoophytorum. Apud Petrum van Cleef, Hagae-Comitum (1766).

[35] Hinrichs S, Patten NL, Feng M, Strickland D, Waite AM (2013). Which environmental factors predict seasonal variation in the coral health of *Acropora digitifera* and *Acropora spicifera* at Ningaloo Reef?. PLoS ONE.

[36] Genikhovich G, Technau U (2009). The starlet sea anemone *Nematostella vectensis*: An anthozoan model organism for studies in comparative genomics and functional evolutionary developmental biology. Cold Spring Harb. Protoc..

[37] Brekhman V, Malik A, Haas B, Sher N, Lotan T (2015). Transcriptome profiling of the dynamic life cycle of the scyphozoan jellyfish *Aurelia aurita*. BMC Genomics.

[38] Lemaire B, Kubota A, O’Meara CM, Lamb DC, Tanguay RL, Goldstone JV, Stegeman JJ (2016). Cytochrome P450 20A1 in zebrafish: Cloning, regulation and potential involvement in hyperactivitiy disorders. Toxicol. Appl. Pharmacol..

[39] Takaku Y, Hwang JS, Wolf A, Böttger A, Shimizu H, David CN, Gojobori T (2014). Innexin gap junctions in nerve cells coordinate spontaneous contractile behavior in hydra polyps. Sci. Rep..

[40] Elphick MR, Mirabeau O, Larhammar D (2018). Evolution of neuropeptide signaling systems. J. Exp. Biol..

[41] Kusserow A, Pang K, Sturm C, Hrouda M, Lentfer J, Schmidt HA, Technau U, von Haeseler A, Hobmayer B, Martindale MQ (2005). Unexpected complexity of the Wnt gene family in a sea anemone. Nature.

[42] Lengfeld T, Watanabe H, Simakov O, Lindgens D, Gee L, Law L, Schmidt HA, Ozbek S, Bode H, Holstein TW (2009). Multiple Wnts are involved in Hydra organizer formation and regeneration. Dev. Biol..

[43] Magie CR, Pang K, Martindale MQ (2005). Genomic inventory and expression of SOX and FOX genes in the cnidarian *Nematostella vectensis*. Dev Genes Evol..

[44] Steele RE, David CN, Techanu U (2011). A genomic view of 500 million years of cnidarian evolution. Trends Genet..

[45] Tarrant AM, Reitzel AM, Kwok CK, Jenny MJ (2014). Activation of the cnidarian oxidative stress response by ultraviolet radiation, polycyclic aromatic hydrocarbons and crude oil. J. Exp. Biol..

[46] Tarrant AM, Payton SL, Reitzel AM, Porter DR, Jenny MJ (2018). Ultraviolet radiation significantly enhances the molecular response to dispersant and sweet crude oil exposure in *Nematostella vectensis*. Mar. Environ. Res..

[47] Petrunak EM, DeVore NM, Porubsky PR, Scott EE (2014). Structures of human steroidogenic cytochrome P450 17A1 with substrates. J. Biol. Chem..

[48] Pallan PS, Wang C, Lei L, Yoshimoto FK, Auchus RJ, Waterman MR, Guengerich FP, Egli M (2015). Human cytochrome P450 21A2, the major steroid 21-hydroxylase: Structure of the enzyme·progesterone substrate complex and rate-limiting C-H bond cleavage. J. Biol. Chem..

[49] Nerbert DW, Wikvall K, Miller WL (2013). Human cytochromes P450 in health and disease. Philos. Trans. R. Soc. Lond. B Biol. Sci..

[50] Guo H, Bao Z, Du H, Zhang L, Wang S, Sun L, Mou X, Hu X (2013). Identification of cytochrome P450 (CYP) genes in Zhikong scallop (*Chlamys farreri*). J. Ocean Univ. China.

[51] Han J, Park JC, Hagiwara A, Park HG, Lee JS (2019). Identification of the full 26 cytochrome P450 (CYP) genes and analysis of their expression in response to benzo[a]pyrene in the marine rotifer *Brachionus rotundiformis*. Comp. Biochem. Physiol. D.

[52] Marlow HQ, Srivastava M, Matus DQ, Rokhsar D, Martindale MQ (2009). Anatomy and development of the nervous system of *Nematostella vectensis*, an anthozoan cnidarian. Dev. Neurobiol..

[53] Twan WH, Hwang JS, Chang CF (2003). Sex steroids in scleractinian coral, *Euphyllia ancora*: Implication in mass spawning. Biol. Reprod..

[54] Tarrant AM, Atkinson MJ, Atkinson S (1999). Estrone and estradiol-17 beta concentration in tissue of the scleractinian coral, *Montipora verrucosa*. Comp. Biochem. Physiol. A Mol. Integr. Physiol..

[55] Goldstone JV, Sundaramoorthy M, Zhao B, Waterman MR, Stegeman JJ, Lamb DC (2016). Genetic and structural analyses of cytochrome P450 hydroxylases in sex hormone biosynthesis: Sequential origin and subsequent coevolution. Mol. Phylogenet. Evol..

[56] Lamb DC, Kelly DE, Kelly SL (1998). Molecular diversity of sterol 14α-demethylase substrates in plants, fungi and humans. FEBS Lett..

[57] Morrison AMS, Goldstone JV, Lamb DC, Kubota A, Lemaire B, Stegeman JJ (2014). Identification, modeling and ligand affinity of early deuterostome CYP51s and functional characterization of recombinant zebrafish sterol 14α-demethylase. Biochem. Biophys. Acta.

[58] Zhang T, Yuan D, Xie J, Lei Y, Li J, Fang G, Tian L, Liu J, Cui Y, Zhang M, Xiao Y, Zu Y, Shang J, Zhu M, Zhan S, Li S (2019). Evolution of the cholesterol biosynthesis pathway in animals. Mol. Biol. Evol..

[59] Nelson MM, Phleger CF, Mooney BD, Nichols PD (2000). Lipids of gelatinous Antarctic zooplankton: Cnidaria and ctenophora. Lipids.

[60] Kanazawa A, Teshima S, Tomita S (1974). Sterol biosynthesis in some coelenterates and echinoderms. Bull. Jpn. Soc. Sci. Fish.

[61] Dermauw W, Van Leeuwen T, Feyererisen R (2020). Diversity and evolution of the P450 family in arthropods. Insect Biochem. Mol. Biol..

[62] Technau U, Schwaiger M (2015). Recent advances in genomics and transcriptomics of cnidarians. Mar. Genomics.

[63] Hasemann CA, Kurumbail RG, Boddupalli SS, Peterson JA, Deisenhofer J (1995). Structure and function of cytochromes P450: A comparative analysis of three crystal structures. Structure.

[64] Werck-Reichhart D, Feyereisen R (2000). Cytochromes P450: A success story. Genome Biol..

[65] Werck-Reichhart, D., Bak, S., & Paquette, S. Cytochrome P450. in The Arabidopsis Book e0028 (2002). 10.1199/tab.0028 PMC3243372 22303202

[66] Baker ME, Nelson DR, Studer R (2014). Origin of the response to adrenal and sex steroids: Roles of promiscuity and co-evolution of enzymes and steroid receptors. J. Steroid Biochem. Mol. Biol..

[67] Markov GV, Tavares R, Dauphin-Villemant C, Demeniex BA, Baker ME, Laudet V (2009). Independent elaboration of steroid hormone signaling pathways in metazoans. Proc. Natl. Acad. Sci. USA.

[68] Altschul SF, Gish W, Miller W, Myers EW, Lipman DJ (1990). Basic local alignment search tool. J. Mol. Biol..

[69] Skinner ME, Uzilov AV, Stein LD, Mungall CJ, Holmes IH (2009). JBrowse: A next generation genome browser. Genome Res..

[70] Kent WJ (2002). BLAT – The BLAST-like alignment tool. Genome Res..

[71] Finn RD, Mistry J, Tate J, Coggill P, Heger A, Pollington JE (2010). The Pfam protein families database. Nucelic Acids Res..

[72] Sigrist CJA, Cerutti L, de Castro E (2010). PROSITE, a protein domain database for functional characterization and annotation. Nucleic Acids Res..

[73] Sievers F, Wilm A, Dineen D, Gibson TJ, Karplus K, Li W, Lopez R, McWilliam H, Remmert M, Soding J (2011). Fast, scalable generation of high-quality protein multiple sequence alignments using Clustal Omega. Mol. Syst. Biol..

[74] ^74^Maddison, W. P., & Maddison, D. R. *Mesquite: A Modular System for Evolutionary Analysis*. Version 3.10 http://mesquiteproject.org (2016)

[75] Stamatakis A (2014). RAxML version 8: A tool for phylogenetic analysis and post-analysis of large phylogenies. Bioinformatics.

[76] Rambaut, A. *FigTree v1.4.3*. http://tree.bio.ed.ac.uk/software/figtree/ (2016)

