# Supplementary material for: The cytochrome P450 (CYP) superfamily in cnidarians
Source: Sci Rep. 2021 May 10;11:9834. doi: 10.1038/s41598-021-88700-y (PMC8110760; doi:10.1038/s41598-021-88700-y)
Supplement: Supplementary file 4 — Supplementary Information 4. [file 41598_2021_88700_MOESM4_ESM.pdf]

Table S4: Distribution of CYP clans in animals and fungi.
Adapted from Nelson et al 2013 with additional data added from this study for *Acropora, Hydra* and Aurelia

	**Clan**											
**Taxa***	**4**	**3**	**mito**	**2**	**51**	**7**	**16**	**26**	**20**	**46**	**19**	**74**
Gnathostomes	+	+	+	+	+	+	+	+	+	+	+	-
Agnathan	+	+	+	+	+	+	-	+	+	-	+	-
Tunicate	+	+	+	+	-	+	+	+	+	-	-	-
Amphioxus	+	+	+	+	+	+	+	+	+	+	+	+
Urchin	+	+	+	+	+	-	+	+	+	+	-	-
Mollusc	+	+	+	+	+	+	+	+	+	-	-	-
Annelid	+	+	+	+	+	+	+	+	+	-	-	-
Insects	+	+	+	+	-	-	+	-	-	-	-	-
Crustacean	+	+	+	+	-	-	+	-	-	-	-	-
Nematode	+	+	+	+	-	-	-	-	-	-	-	-
Nematostella**	+	+	+	+	-	-	+	-	+	+	-	+
**Acropora*** **	**-**	**+**	**+**	**+**	**-**	**-**	**-**	**-**	**-**	**-**	**-**	**-**
**Hydra*** **	**+**	**-**	**-**	**+**	**-**	**-**	**-**	**-**	**+**	**-**	**-**	**-**
**Aurelia*** **	**+**	**+**	**+**	**+**	**-**	**-**	**-**	**-**	**+**	**-**	**-**	**-**
Placozoa	+	+	+	+	+	+	+	-	-	-	-	+
Sponge	+	+	+	-	+	+	+	+	+	-	-	-
Ctenophore	+	-	-	-	-	-	**-**	+	+	+	-	-
Choanoflagellate	+	-	-	-	+	+	+	-	-	-	-	-
Fungi	-	-	-	-	+	?	-	-	-	-	-	-

* Information for the taxa are from Figure 4 Nelson et al 2013 with the exception of clan 16
Clan 16 has been newly designated (Dermauw et al 2020), although in our phylogenetic analysis the CYP16 sequence clustered with Clan26 as did Nematostella in previous analyses
** Nematostella from this study and was included in the Nelson et al 2013 paper as the representative for Anemone
*** From this study
+ Present
- Absent
? Probable lateral gene transfer
