# Supplementary material for: The cytochrome P450 (CYP) superfamily in cnidarians
Source: Sci Rep. 2021 May 10;11:9834. doi: 10.1038/s41598-021-88700-y (PMC8110760; doi:10.1038/s41598-021-88700-y)
Supplement: Supplementary file 5 — Supplementary Information 5. [file 41598_2021_88700_MOESM5_ESM.pdf]

The Cytochrome P450 (CYP) Superfamily in Cnidarians

Kirill V. Pankov[1,2], Andrew G. McArthur[1], David A. Gold[3], David R. Nelson[4], Jared V. Goldstone[5], Joanna Y. Wilson[2,*].

[1] *M.G. DeGroote Institute for Infectious Disease Research, Department of Biochemistry and Biomedical Sciences, DeGroote School of Medicine, McMaster University, Hamilton, Ontario, Canada*

[2] *Department of Biology, McMaster University, Hamilton, Ontario, Canada*

[3] *Department of Earth and Planetary Sciences, University of California Davis, Davis, California, USA*

[4] *Department of Microbiology, Immunology and Biochemistry, University of Tennessee, Memphis, Tennessee, USA*

[5] *Department of Biology, Woods Hole Oceanographic Institution, Woods Hole, Massachusetts, USA*

[*] Corresponding Author: Dr. J.Y. Wilson
Department of Biology, McMaster University
1280 Main Street West, Hamilton ON Canada L8S 4K1 Tel: (001) 905-525-9140 ext 20075
Fax: (001) 905-522-6066
Email: joanna.wilson@mcmaster.ca

Table S1: The complement of 'complete' and 'partial' cytochrome P450 genes in *H. vulgaris, A. digitifera, A. aurita,* and *N. vectensis*. The number of cytochrome P450 genes identified in each CYP gene family. Gene counts include both complete and partial genes. Genes from *N. vectensis* were previously annotated but nomenclature is provided here.

Table S2: The cytochrome P450 fragments in *H. vulgaris, A. digitifera,* and *A. aurita*. The sequence (amino acid) of each cytochrome P450 fragment identified in each genome is listed along with the contig and location (region and start position), length (in amino acids), number of exons, and best BLAST hit on NCBI.

Table S3: The cytochrome P450 families in *H. vulgaris, A. digitifera, A. aurita,* and *N. vectensis*. Species and class where there is likely gene gain or loss are identified

Table S4: Distribution of CYP clans in animals and fungi. Adapted from Nelson et al 2013 with additional data added from this study for Acropora, Hydra and Aurelia. Clan 16 has been newly designated[61] and the table has been updated for all species to reflect this.
